# Sexual behavior of infertile women: a qualitative study

**Published:** 2015-10

**Authors:** Mahshid Bokaie, Masoumeh Simbar, Seyed Mojtaba Yassini Ardekani

**Affiliations:** 1 *Department of Midwifery and Reproductive Health, School of Nursing and Midwifery, Shahid Beheshti University of Medical Science, Tehran, Iran.*; 2 *Department of Midwifery and Reproductive Health, School of Nursing and Midwifery, Reproductive Endocrinology Research Center, Research Institute for Endocrine Sciences, Shahid Beheshti University of Medical Sciences, Tehran, Iran.*; 3 *Addiction and Behavioral Sciences Research Center, Research and Clinical Center for Infertility, Shahid Sadoughi University of Medical Sciences, Yazd, Iran.*

**Keywords:** Sexual behavior, Infertility, Sexuality, Qualitative study

## Abstract

**Background:**

Infertility makes an essential challenge to the sexual life of couples, especially infertile women. When pregnancy does not happen, infertile women think that sexual intercourse is not fruitful and sexual desire became reduce gradually. Infertile women progressively forget that their sexual relationship is also a response to their natural need.

**Objective:**

This qualitative study was conducted to explore the infertility consequences in the sexual behavior of infertile women.

**Materials and Methods:**

This was a qualitative content analysis study; and it was part of a widespread study, used a sequential mixed-method and conducted from August 2014 until February 2015. A purposeful sampling was used to recruit infertile women who had referred to Yazd Research and Clinical Center for Infertility. Data gathering techniques employed in this research included in-depth semi structured open face-to-face interviews and field notes. Credibility, transferability, confirm ability, and dependability were assessed for the rigor of the data collection.

**Results:**

Totally, 15 infertile women and 8 key informants were interviewed. Data analysis showed four themes about impact of infertility on female sexual behavior: 1/ Impact of infertility drugs on couple sexual behavior, 2/ Impact of assisted reproductive technologies on female sexual behavior, 3/ Timed intercourse during infertility and 4/ The psychological impact of infertility on sexual behavior.

**Conclusion:**

Some of Iranian infertile women could cope with their problems, but some of them were very affected by infertility drugs and assisted reproductive technologies procedures. Psychosexual counseling before medical treatment could help them to have a better sexual life.

## Introduction

All women should be entitled to sexual and reproductive health rights. Infertile women are vulnerable for their special sexual and reproductive status (1-3). Having a child is really one of the most noticeable ways of feeling totally pleased from a human point of view, and few couples choose to omit this experience (4). Although one of the key issues about sexual health is relationship between sexual health and infertility, but it seems this aspect of infertility treatment has been ignored in developing countries (5). Regardless of the ICPD's (International Conference on Population and Development) call for supportive sexual health services, in many countries sexual health services are limited to prevention, treatment and care of sexual transmitted illness (STI) and recommendation to use contraceptive methods and condom (6). An estimated number of 48.5 million couples suffer from infertility (7). Around 10.9% of the Iranian couples have experienced infertility during their lifetime (8). Therefore it seems that infertility makes an essential challenge to the sexual life of couples. Infertility management can affect all aspects of lives of infertile women, which can cause many emotional and psychological disorders, such as sexual dysfunction, depression, anxiety, hopelessness, guiltiness, and feelings of worthlessness (9-14). Sexual disorders causing from diagnostic test and medical therapy are common in infertile couples (15), with infertile women more regularly affected than infertile men (16). When pregnancy does not happen, infertile couples think that sexual intercourse is not fruitful, and sexual desire reduces. Infertile couples progressively forget that their sexual relationship is also a response to their natural need (17). Assisted reproductive technologies (ART), can lead to a pregnancy without sexual intercourse. A literature review from Medline Database by Huyghe showed sexual problems are common among infertile couples. So attending to psychological needs of the infertile couples proved important before ART procedures. In some patients, decreased sexual activity, erectile dysfunction and hypoactive sexual desire were increased through the diagnostic and therapeutic procedure (15). Although many researches were conducted about infertility in Iran, there were few studies about sexual behavior of infertile women with qualitative approach in Iran (6, 13, 17-23). The influences of culture on sexual and reproductive behaviors showed that a qualitative study was useful approach to explore the infertility consequences in the sexual behavior (24). The aim of this study was to investigate how infertility influence on sexual behavior and how the medicalization affect sexual behavior in infertile women.

## Materials and methods

**Study design**

This was a qualitative content analysis study; and it was part of a widespread study, which used a sequential mixed-method design (25). This is a standard study method in the social and health sciences that uses a set of techniques to make valid and useable extrapolations from text based on exact comments on coding (26, 27). This study was conducted from August 2014 to February 2015. Qualitative investigators rely on many qualitative approaches to explore and explain the behaviors, attitudes, and life experiences within the context of their lives (28). All infertile female were interviewed in a private area, and approximately each interview continued 30-60 minutes.

**Selection of participants**

In qualitative content analysis, samples were selected purposively and this process continued until data saturation (29, 30). In this study purposeful sampling was used to recruit infertile women who had referred to Yazd Research and Clinical Center for Infertility. Participates were selected with maximal variation regarding duration of infertility, causes of infertility, type of infertility (primary or secondary) and age. Data saturation was obtained with 12 interviews, but three additional interviews were conducted. In addition, eight Key Informant Interviews (KIIs) were chosen including three gynecologists, one nurse, one midwife, one psychologist, one urologist and one psychiatrist.

Inclusion criteria: having infertility record in Yazd Research and Clinical Center for Infertility, two-year history of infertility, speaking Persian, married and interested in participating.

**Data gathering**

Data gathering techniques that employed in this research were in-depth semi structured face-to-face interviews and field notes. Demographic questions were about age, partner age, level of education, job, causes and duration of infertility. Researchers used maximum variation sampling. This is one of the most commonly engaged purposive sampling methods. In this method, participants are chosen purposefully and with a widespread range of variations regarding to the under-study subject (31). Investigators can achieve richer data and an enhanced understanding by choosing participants with various viewpoints and opinions. First of all, directing three individual interviews assisted us to understand the context, to assess the feasibility of the methods and to complete the guide questions. This approach is appropriate for qualitative studies as they are deep and flexible (32). The interviews were focused on the two main following questions:

1. How was your experience about sexual behavior?
2. How drug and ART procedure affect your sexual behavior?

All interviews were recorded with the consent of the participants by a digital recorder. In addition, field notes were written closely after each interview. The field notes protected the primary interviewee responses, as well as the first analytical reflections from the interview content, and any convenient observations that could not be taken by digital recording.

**Data analysis**

The data was analyzed using conventional content analysis method. Three different approaches of qualitative content analysis are conventional, summative and directed. All of these three approaches are used to derive meaning from the content of context and follow to the naturalistic paradigm. In conventional qualitative content analysis, coding groups are gathered directly from the manuscript data (27). This part delivers an impression of main concepts (obvious and hidden content, unit of analysis, meaning unit, compression, abstraction, content area, code, category and theme) associated to qualitative content analysis according to the Graneheim and Landman approach (29).

A main issue when carrying out qualitative content analysis is to pick whether the exploration should emphasis on both manifest (obvious) and latent (hidden) content. Researcher recorded all interviews after every interview and listened to the participant's voice carefully several times, and then the voice was transcribed by "listen N writes" software word by word. At that time researchers read and review context several times. All recorded texts were broken into meaning units. Meaning units are only small parts that are generated from the main text. Concepts of important words and phrases related to the context of the interviews were found. After ending all of the interviews, the researcher team framed concepts into codes. The primary code was designed, each meaning unit was shortened to a “compressed meaning unit” and then, extracted codes were achieved. The research team compared all transcript steps to classify codes relationships and dissimilarities. In the next step, the team discussed about the differences and resolved them and final code was achieved. Similar codes were classified into sub-categories and sub-categories were placed in the main category. Finally the themes appeared. All the audio files and manuscripts are available for review observers.

**Rigor**

Guba and Lincoln (1985) criteria was used for the rigor of the data collection including: 

credibility, transferability, confirmability and dependability (33). In the present study, various characteristics of trustworthiness have been detected. Credibility was settled through length engagement with data, member checking and peer debriefing. Member checking was done by asking four participants to accept the transcripts and developing codes from the interviews. In other hand, member checking was performed by requesting the respondents to check the transcripts and emerging codes from the interviews. Peer debriefing was done by two researcher team member (supervisor and advisor professors). Research team members checked with each other to deal with any doubts in the coding process, sub categories, categories and themes. Investigators individually analyzed the data by classifying and categorizing codes for the participants’ responses to each question, and then the two researchers’ codes and their newest analysis change as themes were compared. In disagreement parts, classifications were clarified and conversation continued until agreement was achieved. Researchers deliberated with each other to contract with any doubts and ambiguities in the coding procedure, classifications and themes. Researchers individually analyzed the data by categorizing and labeling code for the participates’ answers to each question, and then the two researchers’ codes and their newest analysis change as themes were associated. The transferability of this study was confirmed by “rich description and comprehensive reporting of the study process” (34). For transferability (Fittingness), the extracted concepts were given to persons who coordinated with the participants, but did not join the study, to get their judgments about relationship of research results and their own understandings. The main method for founding dependability and confirmability is through checks of the research procedures and results (35). The dependability of this study was conducted by the obvious coding procedure and inter-coder corroboration. Confirmability was obtained by comparing the study results with other evidence and by showing memo and field notes to a next outside investigator (33) “the degree to which the appearances of the data, as suggested by the researcher team, can be established by other investigators (two PhD in sexual and reproductive health) who review and read the study results” was done.

The dependability emphasizes the need for the investigator to explain for the ever-changing framework within which study occurs. It was shown through the investigator’s "research memo" (34).

**Ethical considerations**

The study is one part of the first author’s PhD thesis. Previous to interviews, all participants were made alert of the purposes of the study, and their complete informed consent was gotten based on the Ethics Committee of Shahid Beheshti University of Medical Sciences. Oral and written consents were gained from them for taping and they were assured that the collected data would only be used for research aims. It was also declared to the participants that they could extract from the study anytime they liked and their data would remain confidential during and after the study.

## Results

Totally 15 infertile women and 8 key informants were interviewed. Women were 24-45 years old (34.5±9.5). The education levels in infertile women were from elementary to the MSc degree and in key informant from BS to a specialist. Infertility duration extended from 2-20 (11±9) years and their infertility treatment duration length was 2-19 (10.5±8.5) years. The majority of infertile women (64.70%) had primary infertility. Some other related characteristics of participants are also defined in table I.

**The main themes**

Data analysis showed four themes about impact of infertility on female sexual behavior:

1. Impact of infertility drugs on couple sexual behavior.

2. Impact of ART procedure on female sexual behavior.

3. Timed intercourse during infertility management 

4. The psychological impact of infertility on sexual behavior.

**1. Impact of infertility drugs on couple sexual behavior**

 This theme includes two sub-categories

- Effects of drugs on female desire
- Effects of drugs on male desire

One of the matters mentioned by some of the infertile women was about effects of female infertility drugs on female desire. Some of them thought this drug have a positive impact: "I think these drugs that administrated for stimulating ovulation increase my sexual desire. I do not know, maybe they activate my ovaries." (Female, 34 years old-female infertility).

But some others believed these drugs decrease her sexual desire: "Perhaps, maybe this drug reduced my sexual desire. (Female, 30 years old-female infertility).

Many of them believed drugs have not special effects on their sexuality: "No, this drug did not affect our sexual relationship, but my general condition became worse after injection. (Female, 26 years old- female infertility).

One of our key informants (Gynecologist, 35 years' experience in infertility field) said the female drugs did not increase sexual desire and if it increased in some cases, maybe it is due to positive psychological effects of drugs. In other words, when the person has ovulated, she had a good sense about femininity. 

Some participants believed the infertility male drugs, which were administered for their husbands, influence their sexual desire and sexual cycle: "Since he had injection to improve his sperm count, his sexual desire became better, but his sperm count no difference and his premature ejaculation become worse" (Female, 32 years old-secondary male infertility).

Other key informant (Urologist, 20 years' experience in infertility field) stated androgen drugs improved the male sexual desire, but urologists administrated them in a few cases.

**2. Impact of ART procedures on female sexual behavior**

This theme includes two sub-categories:

- Low frequency of intercourse during ART procedures.
- The negative impact of ART costs and traveling costs on sexual relationship.

Many infertile women believed their sexual intercourse highly were affected during the ART procedure: "Up to 15 days after ART, we did not have sexual intercourse. They said we did not have sexual intercourse until we received ART outcome. If my husband requested to do that, I did not like to have sex, and we did not perform it" (Female, 45 years, male infertility). Not only ART procedure influence on their sexual relationship, but also treatment costs and traveling expenses impressed their sexual activity. "The cost of ART procedures and the cost of traveling was bothering us, made my sexual desire less" (Female, 30 years old, female infertility).

One of the key informants (Midwife, 6 years experience in infertility field) emphasized, although infertility treatment costs in Yazd are lower than other cities, but these concerns influence patients in all aspects of life including sexual life.

**3. Timed intercourse during infertility management**

This theme includes six sub-categories:

- Increasing premature ejaculation problem with a timed intercourse
- No discomfort about timed intercourse by couple and bound to do that
- Negative attitude of female, male or both about timed intercourse
- Low female desire during the times less probability of pregnancy
- No recommendation for timed intercourse by physician
- Improving couple relationship after removing timed intercourse

In some infertile men premature ejaculation became worse during timed intercourse: "From the first, my husband suffered from premature ejaculation. Whenever we had timed intercourse, he had more trouble. (Female, 44 years, male infertility). "Premature ejaculation is common in the population and it is increased after diagnose infertility" (Psychiatrist, 25 years' experience in infertility field).

For some of the participants, it was important to perform a doctor^'^s order and bound to do that. "Because the doctor told us to have timed intercourse every two nights, if we did not have interest to do that, we had to do it. It is not our concern, it was not so long" (Female, 34 years old, female infertility). "We were not upset about timing intercourse. We want a baby and we must do physician order" (Female, 43 years old, male infertility).

Another concern of infertile couple was interfering of timing intercourse. "My husband hated this timed intercourse. He thought I did not love him and my aim to intercourse was just to get pregnant" (Female, 45 years old, male infertility). "I was nervous because we must have sex every two nights" (Female, 40 years old, female infertility).

Some educated participants do not prefer doing intercourse after ovulation period: "I tried to do intercourse around ovulation period. After this time, the probability of pregnancy becomes low and I do not prefer sexual intercourse during this time" (Female, 45 years old, male infertility).

Another participant mentioned that the doctor had not ordered them timed intercourse: "My physician had not told us when we must perform intercourse" (Female, 32 years old, male infertility).

Some of the participants emphasized their relationship become well after removing the time schedule: "Since we had not timed intercourse, our relationship becomes better" (Female, 45 years old, male infertility).

One of key informant (Urologist, 20 years' experience in infertility field) said most of patients do not upset from time intercourse because most of them are young and have 2-3 times intercourse weekly.

**4. The psychological impact of infertility on sexual behavior**

This theme includes:

- The impact of infertility on self-efficacy and confidence Infertility as a cause of depression
- Offensive thoughts during sex
- Little impact of infertility on sexual behavior
- Blame the partner for cause of Infertility
- Stressful process of ART

The majority of participants and key informants believed that infertility had major effects on the physiological status. Many participants highlighted that their self-efficacy and confidence became less after infertility diagnosis: "I think that infertility affects my confidence and decreases my self-confidence and self-esteem" (Woman, 34 years old, female infertility). "Most of infertile women reported low self-efficacy and confidence. They became depressed after ineffective treatments (Psychologist, 18 years' experience in infertility field).

Some of them said Infertility made them depressed: "After recognizing the infertility problem, I got nervous. Every day I think about this condition and I became depressed" (Woman, 27 years old, male infertility).

Few of them suffer from offensive thoughts during sex: "Many times during sex, annoying thoughts surrounded me, I try to keep them away and after 5-6 minute I come back to relationship" (Woman, 27 years old, male infertility).

Many of infertile women confirm that infertility had a little effect on their sexual behavior: "My husband and I have a good sexual desire, most of the time infertility did not have a negative impact on our sexuality" (Female, 34 years old, female infertility).

Three participants with male factor infertility blame their partner for Infertility cause: "Sometimes during sex I told him: you are the reason why we do not have a child" (Female, 45 years, male infertility).

Most of infertile women stated ART was very stressful: "Well we can't deny it that infertility is very stressful especially during ART" (Female, 45 years old, male infertility).

Many of key informant believed infertility and ART were very stressful and sexual and psychological consulting before ART is very useful. (Gynecologist, 20 years' experience in infertility field) (Gynecologist, 35 years' experience in infertility field) (Nurse, 25 years' experience in infertility field).

**Table I:** Participant's and key informant's characteristics

**Participant's characteristics**	**n (%)**
**Age (years)**	11 (73%)
<35	4 (27%)
≥36	
**Job**	
Housewife	13 (87%)
Employed	2 (13%)
**Type of infertility**	
Primary	13 (87%)
Secondary	2 (13%)
**Causes of infertility**	
Female	8 (53%)
Male	7 (47%)
Both	0
**Females’ education status**	
Postgraduate	1 (7%)
Graduate	6 (40%)
High School Diploma	5 (33%)
Pre-Diploma	3 (20%)
**Males’ education status**	
Postgraduate	0 (0%)
Graduate	5 (33%)
High School Diploma	6 (40%)
Pre-Diploma	4 (27%)
**Duration of infertility (years)**	
2-4	3 (20%)
5-10	9 (60%)
>11	3 (20%)
**Duration of marriage**	
Up 10 years	9 (60%)
>11 years	6 (40%)
**Parental status in couples with secondary infertility**	
No Children	14 (93%)
One Child	1 (7%)
**Key informant (degree)**	**No of years' work in infertility field**
Gynecologist 1 (Professor)	35
Gynecologist 2 (Professor)	20
Gynecologist 3 (Associated professor)	12
Midwife (Bachelor of Science)	6
Psychiatrist (Professor)	25
Nurse (Bachelor of Science)	25
Urologist (Associated professor)	20
Psychologist (Master of Science)	18

**Table II:** The main themes and sub- themes in this study (Sexual behavior in infertile women)

**The main themes**	**Sub-themes**
**Impact of infertility drugs on couple sexual behavior**	
	Effect of female infertility drugs on female desire
	Effect of male infertility drugs on male desire
**Impact of ART procedures on female sexual behavior**	
	Low frequency of intercourse during ART procedures
	Negative impact of ART costs and traveling costs on sexual relationship
**Timed intercourse during infertility treatment**	
	Increasing premature ejaculation problem with a timed intercourse No discomfort about timed intercourse by the couple and bound to do that Negative attitude of female, male or both about timed intercourse Low female desire during the times less probability of pregnancy No recommendation for timed intercourse by physician Improving couple relationship after removing timed intercourse
**The psychological impact of infertility on sexual behavior**	
	The impact of infertility on self-efficacy and confidence Infertility as a cause of depression Offensive thoughts during sex Little impact of infertility on sexual behavior Blame the partner for infertility cause Stressful process of ART

ART: Assisted reproductive technologies

## Discussion

The aim of this study was to investigate how infertility influence on sexual behavior and how the medicalization affect sexual behavior in infertile women. Research results indicated that infertile women are affected by infertility treatment. Although some literature reviews show infertility does not necessarily lead to sexual problems or bad sexual relationship, but many of them confirmed that psychosexual disorders were present (13, 23, 36-39). 

Hasanian *et al*, exhibited that infertility was the essential cause of reduced sexual function within four to six years after infertility in Egypt (40). According to a study in the Gambia, where marital permanency is by this time a concern, infertility is seen as a main threat to marital permanency in developing countries (41). There is a significant difference in the experience of infertility in developed and developing countries (42). 

In developed countries, having children is voluntary (43), but this in developing countries is compulsory and most of girl trained for motherhood (44), therefore there are direct psychosocial consequences on infertile women (45). The first extracted theme of this qualitative study was "Impact of infertility drugs on couple sexual behavior". Analysis of participants’ experiences showed side effect of female infertility drugs distributed many of the participants. Medical treatment had different effects on infertile women desire, but had a positive effect on male desire (46). Sexual disorders resulting from diagnosis and medical therapy is common in couples with fertility problems, with women more frequently affected than men (47).

One of the important findings was low infrequent coitus among Indian infertile couples (48). Greil *et al* in their review article stated that most studies did not pay attention for sexual and psychological infertility effects (43). Currently, more than a few studies have shown that besides medical approach, it is essential to pay attention to the other aspects of infertile patients' lives (15, 49, 50). According to the Lechner study, infertility therapy, treated infertile women in the psychosexual domain (51). 

Ramezanzadeh confirmed sexual desire and satisfaction in men after infertility diagnosis, regardless of the cause of infertility were reduced (52). But male infertility therapy was different from females. Some researches confirmed that androgen supplements therapy improved the androgen deficiency-related symptoms and enhanced the mental state and quality of life of infertile men and sexual desire (53, 54). Nearby to 70% of infertile men using testosterone improved spermatogenesis within 6 months of Testosterone discontinuation (55). 

Ramasamy and Lipshultz showed testosterone deficiency syndrome and erectile dysfunction in young infertile men seems not to be depended on hormone levels (55). Although, most studies have not divided the psychosexual consequences of infertility from its interventions or treatments, but low sexual coital frequency and low sexual desire in infertile women were reported in some of them (48, 51, 52). Despite of the cause of infertility, women were more affected from infertility treatment than men, therefore sexual counseling before infertility treatments advised (57).

The second extracted theme was "impact of ART procedure on female sexual behavior". Iranian infertile women were hoping to be cured by the ART procedure (58). Complications and variety of infertility treatment affect the sexual relationship and frequency of intercourse (13). ART is a multidimensional traumatic treatment; all of the participants believed ART procedures were lengthened, stressful and tedious. They did not prefer to have sex with their partner until receiving the answer. After this time if the answer was negative they did not prefer to have sex near 1-2 month (59). These approaches took emotionally challenges to infertile couples. Women who seek infertility treatment have been described to be more emotionally distressed and nervous than women in the general population (60-61). Verhaak *et al* results in one of the largest review study showed that the difference in emotional adjustment is mild at the onset of the study, while unsuccessful ART procedures intensifies negative emotions like anxiety and depression (62), which persist after consecutive unsuccessful cycles. In our study, most of the participants emphasized low sexual desire during ART therapy and until they received the outcome of their treatment. Overall, most infertile women fail to acceptably adapt ART procedures. However, a significant number of participants show clinical psychological problems (62). Infertile women reported more variations in their sexual relationships. Sexual relationships were negatively affected among them (51). Health was even more obviously affected in women with previous unsuccessful in-vitro fertilization experience (37). In our study, most of the women reported their intimacy was greater during treatment. Ohl *et al* showed that both infertile partners keep a respectable relationship and support each other, but many infertile couples sense sexual desires reduction during treatment (49). Reder *et al* studied the impact of infertility, medical care and ART on sexuality and marital relations. Results showed that both infertile partners keep a good relationship and support each other. But, whereas enjoyment during sexual intercourse is not affected much, many infertile couples feel a reduction of their sexual desires. Different factors, such as the type of protocol, duration (years) of ART, age, sex, and type of infertility can effected on their sexual relationship (63). All of these studies support our results, although sexual intercourse might be decreased during treatment, but it seems supportive health system could help infertile couples during the ART procedure.

The third extracted theme was "Timed intercourse during infertility treatment". Our findings about timed intercourse had many variations, in other words, some of them hate timed intercourse and some of them accept it easily. In many male partners premature ejaculation was becoming worse after this timetable schedule. But some of them were happy from this timed intercourse and they performed it calmly. The timing of sexual intercourse powerfully enhanced the chance of pregnancy, although the real number of fertile days in a female's menstrual cycle is ambiguous (64). For an increasing fertility chance most of the specialists recommended timed intercourse for infertile couples for increasing chances of pregnancy (65), with this program, sexual relationship has become like a duty and obligatory action rather than a pleased and joyful task (66). One of Kohan concept was “discouragement of sexual relations”. Most of her participants said they did their sexual relationship with the aim of pregnancy after infertility diagnosis, and as they did not achieve child, they had no desire for sexual relationship anymore and found it a hopeless action, so they avoided that (23). Although many studies confirmed this program can decrease sexual desires, and caused unpleased sex (15, 23, 63), near half of our participants had no discomfort from timed intercourse. They explained their feeling, first this timed intercourse was not so long and female sexual desire was high at ovulation time. Second, they understood this program improved their chance to achieve a baby.

The fourth extracted theme was "the psychological impact of infertility on sexual behavior". The results showed that most of infertile women were suffering from infertility more than their husbands, and this issue was not related to type of infertility (22, 67, 68). There are many evidences that show the stress caused by infertility has a direct effect on psychometric parameters in infertile couples (13, 15, 38, 50, 53, 69-73). 

Infertility can cause sexual dysfunction, unhappiness, anxiety and social isolation (51, 74, 75). 

Chachamovich *et al* in their systematic review confirmed that infertile women were more affected by infertility than their partners. Duration of the infertility, educational level, tendency to childbearing, poor marital relationship and previous ART history were predictors of lower mental health scores in infertile men (76, 77). Shindel *et al* reported that erectile dysfunction, depression and sexual relationship problems were dominant among male infertile couples and they reported significantly lower scores on mental health (78). 

Infertility had closely influenced on sexuality, self-esteem and self-image. Sexuality is also essential means of stating feelings of intimacy and closeness in partnership. During infertility therapy, the enjoyable experience of sexual intimacy may be adversely affected and this may help marital distress (79). A phenomenological study about life experience with infertility by Khodakarami showed that infertility, affects the sexual relationships and emotional status in infertile couples (13, 22). 

As a systematic review by Gameiro *et al *exposed, the psychological burden stemmed from the infertility treatment has been one of the highest reasons for the stoppage of infertility treatment (80). Fear of receiving pregnancy result and how tell the possible negative result to her husband in some participants reported by Hasanpoor (13). Concern about receiving a pregnancy test, were common in our participants. This finding was confirmed by others (70, 72, 81). Although psychological problems during infertility were increased, but it teach them to be friendly in life and change their sexual life better (63). 

Childbearing in the Eastern cultures is one of the main values of family and when the woman is childless, probable psychological crisis increase (82). The study finding showed infertile women were more affected by infertility in both cause of infertility (male or female factor). Becoming mother is very important for Iranian women. Expensive and grueling infertility treatments in one hand and a failed attempt to achieve children in other hand, is associated with low frequency of intercourse and sexual pleasure. It leads to doing sex as a duty to satisfy the husband. One of our interesting findings was, some women blamed their partners for male factor Infertility, but none of them with female infertility had been charged by husbands. Researchers could not find any evidence with this subject. It might show that young infertile men supported their spouses. The highest level of male education was found in three cases.

## Conclusion

Medicalizations have a various affect in Iranian female sexual behavior. Some of them could cope with their infertility problems, but some of them were very affected by infertility drugs and ART procedures. Psychosexual counseling before medical treatment could help them to have a better sexual life. Enhanced collaboration between infertility specialists and psychologist and sexologist would definitely help these couples to preserve a better sexual life and quality of life in general and probably also improve the quality and the results of infertility treatment. The engaged participants included infertile women who volunteer to participate in this study compared with non-volunteered women, may have different sex-psychological outcomes.

## Note

***This article extracted from PhD thesis (Mahshid Bokaie)***
